# Modulated Acquisition of Spatial Distortion Maps

**DOI:** 10.3390/s130811069

**Published:** 2013-08-21

**Authors:** Alexey Volkov, Jerneja Žganec Gros, Mario Žganec, Tomaž Javornik, Aleš Švigelj

**Affiliations:** 1 Alpineon d.o.o., Ul. Iga Grudna 15, Ljubljana 1000, Slovenia; E-Mails: jerneja.gros@alpineon.si (J.Ž.G.); mario.zganec@alpineon.si (M.Ž.); 2 Jožef Stefan Institute and Jožef Stefan International Postgraduate School, Jamova c. 39, Ljubljana 1000, Slovenia; E-Mails: tomaz.javornik@ijs.si (T.J.); ales.svigelj@ijs.si (A.Š.)

**Keywords:** 3D vision, range sensors, triangulation, laser scanning, modulation, CDMA

## Abstract

This work discusses a novel approach to image acquisition which improves the robustness of captured data required for 3D range measurements. By applying a pseudo-random code modulation to sequential acquisition of projected patterns the impact of environmental factors such as ambient light and mutual interference is significantly reduced. The proposed concept has been proven with an experimental range sensor based on the laser triangulation principle. The proposed design can potentially enhance the use of this principle to a variety of outdoor applications, such as autonomous vehicles, pedestrians' safety, collision avoidance, and many other tasks, where robust real-time distance detection in real world environment is crucial.

## 1. Introduction

Over the recent years, significant research effort has been spent to introduce the third dimension to computer imaging. In customer electronics, these efforts have already led to creation of new products, new markets and opportunities: gesture control is becoming a “must have” feature of forthcoming home entertainment systems, revealing the new concepts of *human-to-machine interfaces* (HMI) [1–3]. For the industry, medicine, security and even archeology 3D vision systems are already there: surface quality control, non-invasive reconstruction of cultural heritage, reverse engineering, surveillance and face recognition systems—all of these applications utilize *3D imaging sensors* or *range sensors* [4].

As well as conventional cameras, range sensors are equipped with a light-sensitive element—either a standard or a specially designed CMOS or CCD sensor [5–7], but whereas classical cameras produce images or *bitmaps* representing the light intensity distribution over the scene, this class of devices produces *range maps*—a digital representation of the scene surface based on information about the distances from the sensor to the objects of the scene.

Several techniques have been proposed for range measurement [8–16]. They may be categorized in two main categories: *active* and *passive*. Passive methods, such as *stereo-vision* [17] and *shape-from-focus* [18], do not use any special source of electromagnetic radiation for extracting the range information, while active range sensors illuminate the scene with some type of structured light in visible or infra-red spectrum with further reconstruction of 3D structure of the scene by measuring the temporal or spatial distribution of the reflected signal (*time-of-flight* [19], *interferometry* [20]).

Miniaturization and increasing performance of CPU-s, plenty of cost-efficient and powerful *systems-on-chip* (SoC-s) and embedded platforms, intensive evolution of CMOS sensors make forthcoming computer vision systems promising for solving a variety of *simultaneous localization and mapping* (SLAM)-related tasks for robotics and autonomous vehicles. But there are still some challenges which should be solved before we can rely on these systems in real world environment. For outdoor scenarios, ambient light and attenuation of the transmitted signal due to the atmospheric effects cause problems in most of the existing techniques of range measurement [21]. Furthermore, in case when several active sensors scan the same area simultaneously, they may experience *mutual interference* problems.

In this paper we propose the solution which solves these problems—*modulated acquisition of projected patterns*. Reflected from the scene as a *spatial distortion map*, such patterns are utilized as robust raw data for further 3D reconstruction of the scene in real time. The discussed solution does not represent another pattern-based 3D scanning method; instead we are focusing on the robustness of data acquisition in order to improve the input for numerous existing range measurement approaches. The desired robustness is not achieved by any kind of spatial encoding like pattern, shape or color as suggested in some of the previous studies [22–24], but by signal encoding in the time domain.

The paper is organized as follows: in Sections 2 and 3 the physical principle and the concept of the proposed solution are discussed. The outline of the design is given in Section 4. In Sections 5 and 6 the proposed performance evaluation methodology is discussed. In Section 7 the proof of feasibility is demonstrated for the proposed approach. Finally, the concluding remarks and further work are given in Section 8.

## 2. Laser Triangulation Principle

**Figure 1.**
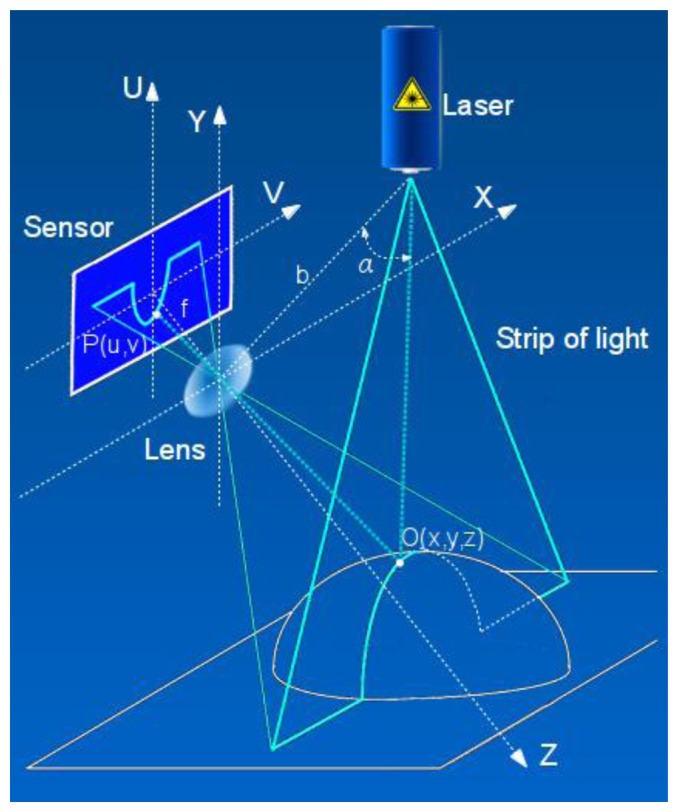
Laser triangulation principle.

The *active triangulation system* (ATRIS) 3D imaging approach developed in our group is based on laser triangulation [25], one of the most well known optical range measurement approaches. Its schematics are depicted in Figure 1.

In this scenario, a laser strip is projected onto a measurement object. The reflected light falls onto a receiving element (sensor). The point *O*(*x*,*y*,*z*) belonging to the projection at the object's surface is reflected at position *P*(*v*,*u*) on the sensor's plane depending on the distance to the object and the angle *α* between the base line and the plane of the projected light. The base line connects the optical center of the lens and the laser source. Given the lens' focal length *f* and the length of the base line *b*, the coordinates of the original point *O*(*x*,*y*,*z*) can be calculated by the Equation (1):

(1) $$\left[\begin{array}{c} x\\ y\\ z \end{array}\right]=\frac{b}{f\;\text{ctg}\left(\alpha \right)-u}\left[\begin{array}{c} v\\ u\\ f \end{array}\right]$$

The above algorithm is then repeated for all points of the reflected profile, while the laser strip is scanned across the object. In this way a dense range map representing a shape of the object can be acquired.

This method of range measurement is appropriate for indoor applications such as profilometry or surface quality control. It is affordable since it does not require any expensive high-frequency circuitry needed for *time-of-flight*; it is relatively robust since it is not based on quantification of accumulated charge, *i.e.*, brightness of pixel. Instead, it relies on identifying the coordinates of the brightest pixels. However, in circumstances of intricate ambient light it is rather tricky to distinguish the pixels illuminated by the laser from those illuminated by the external light sources.

It is obvious that ambient light can always be overpowered by the laser, although this is strongly undesirable because of high energy consumption and eye-safety concerns. In order to improve the robustness of the captured data, the concept of *modulated laser triangulation* has been introduced in [26] by our research group, where we have proposed several possible designs of range detectors utilizing the sequential frame acquisition controlled by random binary sequences. In this paper we are demonstrating appropriate binary sequences which guarantee the immunity to environmental factors and propose the methodology for evaluation of the system performance.

In order to accelerate the acquisition of raw data required for 3D reconstruction, a multi-line pattern or another complex pattern can be projected onto a scene. Its reflection is then acquired as a *spatial distortion map* in a single snapshot instead of scanning the scene by a single line step-by-step. This improvement coupled with modulated acquisition of spatial distortion maps makes the laser triangulation method promising for outdoors applications, such as collision avoidance, autonomous navigation, *etc.*

## 3. The Concept of Modulated Acquisition of Projected Patterns

The performance of the proposed system mainly depends on the ability to capture a sharp and accurate image of the projected pattern. The better the contrast between the projected pattern and background; less time will be required to detect the projected pattern in the snapshot. Missing parts of the pattern or excess lines not belonging to the projected pattern can lead to erroneous results in the range map.

The problem of distinguishing the projected pattern from the background and interfering projections at the sensor's view area appears to be very similar to the *general receiving problem* [27] in multiuser communication systems, where multiple transmitter-receiver pairs communicate simultaneously over the same channel. Each receiver in such system should be able to filter out the data addressed to it individually from the observed signal containing data sent to other users.

In the *direct sequence spread spectrum* (DSSS) scheme, the problem of user separation at the receiver side is solved by multiplying the data stream transmitted to a certain user by the pseudo-random periodic sequence of {−1;1} known as *signature* or *spreading code*. The frequency of this periodic signal is much higher than the frequency of the original data stream. Thus, the energy of the original signal becomes spread across the whole band of transmitted noise-like signal; hence the name of this technique is *spectrum spreading*. To reconstruct the original data, the receiver multiplies the observed noise-like signal by the personal signature, which yields in raising the level of original data constituent above the noise and the interfering signals. This effect of *signal-to-noise ratio* (SNR) improvement is known as *processing gain*. Signals addressed to other users are modulated with different signatures; they produce no processing gain at the given receiver and can be easily suppressed along with the channel noise. This is the essence of *code division multiple access* (CDMA) communications [28]. As shown in recent research results [29–31], the performance (*user capacity, bit error rate, SNR*) of CDMA systems is mainly determined by the correlation properties of spreading codes.

In the following sections we show that a similar effect of “selective perception” is also achievable with imaging. By applying the technique of *direct sequence modulation* to sequential image acquisition while illuminating the scene with the source of light modulated by the same sequence, we can capture *only* the reflection of the modulated illumination pattern without the influence of other sources of illumination. The captured reflection carries the information about the spatial distortion of the projected pattern needed for range measurement. The interfering projections as well as general (background) illumination are eliminated from the acquired image due to the nature of CDMA system.

## 4. Experimental Setup

An attractive solution for implementing an imaging system described in Section 3 is the *dual-output* or *gated* CMOS sensor [32,33]. Each pixel of such sensor has a single photodiode with duplicated charge storage and readout circuitry. Storages can be switched at the stage of integration by the external modulation signal of considerably high frequency, which can be simultaneously used as a laser strobe. Using this technique, we could have two separate images captured during the same exposure time: one with enabled laser and another with disabled laser. At the readout phase, one image is subtracted from another, resulting in image containing only the difference induced by the laser illumination. Currently, the only disadvantage of this approach is poor availability of gated sensors.

**Figure 2.**
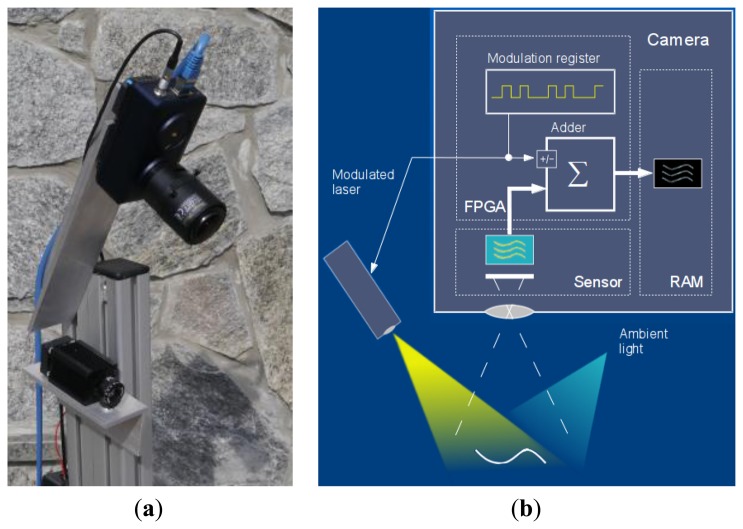
Experimental setup: (**a**) hardware implementation and (**b**) block scheme.

To prove the concept of modulated acquisition, we have built our experimental setup (Figure 2) around a fast Velociraptor (Optomotive mechatronics Ltd, Ljubljana, Slovenia) industrial camera [34] based on high-speed conventional commercially available non-gated CMOS sensor CMOSIS CMV2000 and FPGA processing core. Instead of performing “in-pixel” demodulation, the demodulation logic has been programmed in FPGA: conditional adder and modulation register were introduced at the data path from the sensor to RAM.

The camera makes series of snapshots at high frame rate (up to 600 fps) with short exposure time. Each time a snapshot is taken the laser is turned ON or OFF according to the control (highest) bit of pseudo-random binary sequence *c* ∈ {0;1} of length *N* being cyclically shifted in modulation register. Through the full cycle of the sequence (*frame time*), each pixel of the currently captured image (*sub-frame*) is conditionally added to the cumulative sum of corresponding pixels of the previously captured sub-frames. If the control bit is 1 (laser is enabled) the pixel value is added to the sum, otherwise (if laser is disabled) it is subtracted respectively.

## 5. Signal and Noise Evaluation

As defined above, each frame in our ATRIS 3D system is built of cumulative per-pixel sum of *N* sub-frames taken with positive or negative signs according to the control bit of the modulation sequence. Unlike in communication systems where a receiver extracts the transmitted signal from the observed noise-like temporal signal, in our case “signal” and “background noise” are two spatially combined groups of pixels. To build the range map, we must find the coordinates of “signal” pixels in captured images.

**Figure 3.**
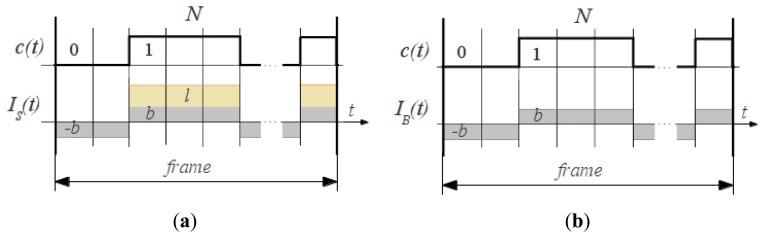
Background noise suppression: pixel intensity through the frame cycle for (**a**) illuminated and (**b**) background pixels.

In the following we evaluate the intensity of the “signal” pixel in comparison to all the rest (background) pixels regarding the applied modulation scheme. As depicted in Figure 3, for the “signal” group of pixels a constant background intensity *b* is present through the whole frame integration cycle, while the laser illumination intensity *l* is added to the resulting pixels intensity *I_S_* only when *c*(*t*) = 1 (Figure 3a). For the “background noise” group only the background intensity *b* is present through the integration cycle of the resulting pixel intensity *IB* (Figure 3b). Let us analyze the resulting intensity for the both groups of pixels according to the methodology known from CDMA communications.

### 5.1. Background Pixels

Before proceeding with the analysis, consider the following transform *v*(*i*) of the original modulation sequence *c*:

(2) $$v(i)=-{\left(-1\right)}^{c_{i}}=\left\{ \begin{array}{l} +1,c_{i}=1\\ -1,c_{i}=0 \end{array}\right.$$

Than the resulting intensity of the background pixels *IB* can be formalized as follows:

(3) $$I_{B}=\sum_{i=0}^{N-1}(\bar{b}+\Delta b_{i})v_{i}=0+\delta$$

Obviously, for the *balanced* sequence *c* (the number of zeros is equal to the number of 1-s) the mean background light intensity *b̄* is completely eliminated. The resulting intensity will hold only some uncompensated remainder *δ* of the background light deviations Δ*b_i_* tending to zero when these fluctuations are relatively slow in comparison to sensor's frame rate.

### 5.2. Illuminated Pixels

Now we shall estimate the intensity of illuminated pixels *I_S_*. We can assume that the laser intensity *l* is constant for all the positive chips of modulation sequence (*c*(*i*) = 1). Then the resulting intensity of illuminated pixels is the following:

(4) $$I_{S}=\sum_{i=0}^{N-1}(\bar{b}+\Delta b_{i}+lc_{i})v_{i}=\frac{lN}{2}+\delta$$

As in Equation (3); the constant constituent is eliminated and only the sum of *l* coinciding with the positive chips of *v* remains. Again; the uncompensated remainder of background light deviations tends to zero in circumstances of the constant or slowly changing intensity of the external illumination. Hence; using *balanced* sequence *c* the resulting pixels intensity equals to *l* multiplied by the number of ones in modulation sequence (*N*/2) plus the influence of uncompensated background light *δ*.

### 5.3. System Characteristics in Terms of CDMA Performance

Now we can characterize our system in terms of *processing gain* (Equation (5)) and *SNR* (Equation (6)):

(5) $$PG=\frac{I_{S}}{l}=\frac{lN+2\delta }{2l}$$

(6) $$\text{SNR}=\frac{I_{S}}{I_{B}}=\frac{lN}{2\delta }+1$$

From the above analysis we can expect how the demodulated image will look like: bright image of the intersections of the projected pattern with the object's surface against the completely black background—exactly what we expect from the spatial distortion map. A projected pattern will be perceived much brighter than it would appear without modulation. High contrast with the black background makes the detection of projected pattern quite simple.

For static scenes, high *SNR* is expected since *δ* tends to zero. For dynamic scenes, some “gray trails” left by the edges of moving objects could be noticed. We can suppress these artifacts by setting the threshold while detecting the projected lines. Increasing the sensor's frame rate will also lead to noise reduction since the integrated frame will accumulate fewer deviations.

## 6. Mutual Interference Compensation

As mentioned in Section 2, immunity to mutual interference is one of the key features of CDMA systems. This immunity is determined by the cross-correlation properties of spreading codes. In telecommunication systems, a number of known families of spreading codes exist, each solving a specific task of separating signals in common channels. Orthogonal Walsh-Hadamard codes [35] are widely used for synchronous CDMA systems, such as downlink of CDMA2000, where a single transmitter (a base station) takes care of phase synchronization of the transmitted signals. Unfortunately, not all of them preserve orthogonality when shifted against each other. Other known families of codes are *Gold codes* [36] and *m-sequences* [37]. They are appropriate when synchronicity between the multiple transmitters cannot be established (uplinks, CW-radars). In this case at least the deterministic *signal to interference ratio* (SIR) should be guaranteed for all the users of the system.

The “receiver” and “transmitter” of our system are synchronized by design, since the sensor and the laser are integral parts of the same unit. However, we cannot synchronize multiple range-finder units which may appear operating on the same place. At any moment an interfering projection may appear at the given sensor's view area, and we should be able to filter it.

A similar problem has been studied in [38], where several sensors are connected into a network. These sensors “wake-up” and transmit the captured data asynchronously. In order to avoid MAI (*multiple access interference)*, the authors proposed a set of *cyclic orthogonal codes* for spreading. These codes were derived from Walsh-Hadamard matrix of size *N* × *N*, where *N* = 2*^k^*. As found from observations, the rows of such matrix can be combined into some finite number of non-unique sets of *k* + 1 rows which preserve mutual orthogonality at any phase shift. Taken as spreading codes, they can safely accommodate *k* + 1 users of asynchronous CDMA system.

Similarly, we can find appropriate subsets of codes meeting the requirement determined by our design: we need zero *periodic correlation* of given user's modulation sequence with another user's sequence represented in original unipolar form, as it is used to strobe the laser. For further analysis, we consider that interfering devices are identical, *i.e.*, having the same intensity of laser illumination *l*, the same exposure time and the same length of modulation sequence *N*. Then the above criteria could be formalized as follows:

(7) $$R_{v,u}\left(\tau \right)=\sum_{i=0}^{N-1}v_{i}u_{i+\tau }=0,\forall \tau$$

where *v* is a given user's modulation sequence in bipolar form {−1;+1}, *u* is an interfering user's modulation sequence in unipolar form {0;1} and *τ* is a cyclical phase shift. Considering *u* and *v* as continuous periodic functions having the same period *T* and delay *τ* between each other, the above criteria could also be presented like this:

(8) $$R_{v,u}\left(\tau \right)=\int_{0}^{T}v(t)u(t+\tau )dt=0,\forall \tau$$

Since our design is limited to a maximal sequence length of 32 bits, we searched the Walsh-Hadamard matrices of size from 4 × 4 to 32 × 32 with the quite simple algorithm and have found some regularity in distribution of rows fulfilling the criteria (7). We have also found that from a *N* = 2*^k^* size matrix one can choose only *k* rows meeting our requirement. The first row is never selected since it contains only +1-s. The second row is always selected because it contains the repeated pattern of {−1;+1} which can be combined with any other. Then the 3^rd^ or 4^th^ row can be selected. After that for each *k* from 3 to given size of matrix 2*^k^* one additional row could be chosen from the range of [2*^k^* − 2*^k^*/2 + 1, 2*^k^*] in combination with previously selected rows.

For example, to obtain a desired set of cyclic orthogonal codes with length 8, one can select only three rows in possible combinations like 2, 3, 5; 2, 4, 7; 2, 4, 8, *etc.* (Table 1).

Now let us introduce the interfering illumination to Equation (3) of the background noise expressed as a continuous periodic signal:

(9) $$I_{\text{MAI}}\left(\tau \right)=\int_{0}^{T}\left(b(t)+l_{\text{MAI}}u(t+\tau \right))v(t)dt=0+\delta ,\forall \tau$$

**Figure 4.**
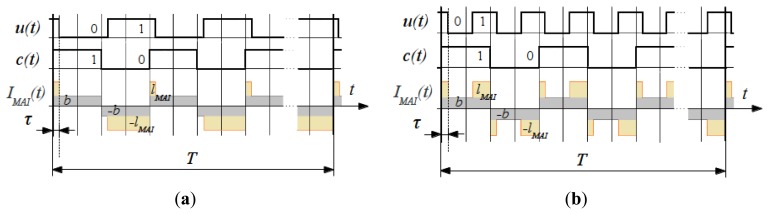
Mutual interference suppression: interference pixels intensity through the frame cycle for a pair of (**a**) phase-shifted non-cyclic orthogonal codes and (**b**) cyclic orthogonal codes.

With properly selected code sets, the interfering illumination *l_MAI_* is completely suppressed at any delay *τ* according to Equation (8). Background noise *b*(*t*) is eliminated as well, since Walsh-Hadamard codes are balanced. Graphical representation of mutual interference compensation is depicted in Figure 4.

It can be seen that in case when *u* and *c* are cyclic orthogonal, the interfering illumination intensity *IMAI* has been eliminated.

## 7. Experimental Results and Discussion

We have tested our setup in several scenarios, involving strong jamming, direct sunlight and mutual interference. For 16-bit long modulation sequence and sensor's frame rate of 480 fps we have achieved a stable live view of reflected patterns at 30 fps. The snapshots in the following figures were taken in plain and demodulated live view modes. The scene was illuminated using modulable 650 nm LED laser combined with a DOE (diffractive optical element) projecting a pattern of parallel horizontal lines.

**Figure 5.**
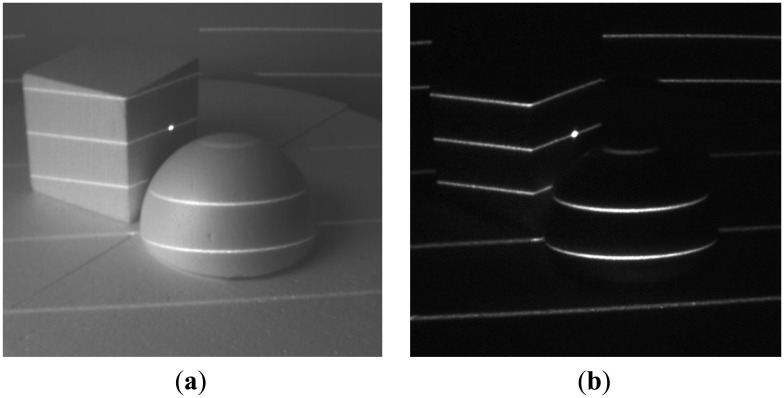
Background noise suppression: (**a**) plain image; (**b**) demodulated image.

**Figure 6.**
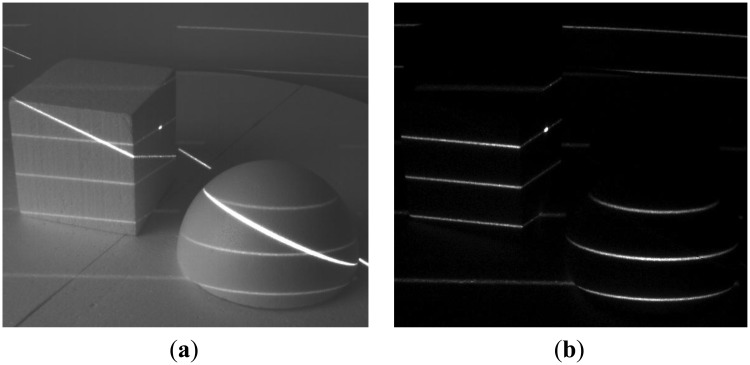
Interference suppression: Strong continuous laser line interference. (**a**) Plane image; (**b**) demodulated image.

**Figure 7.**
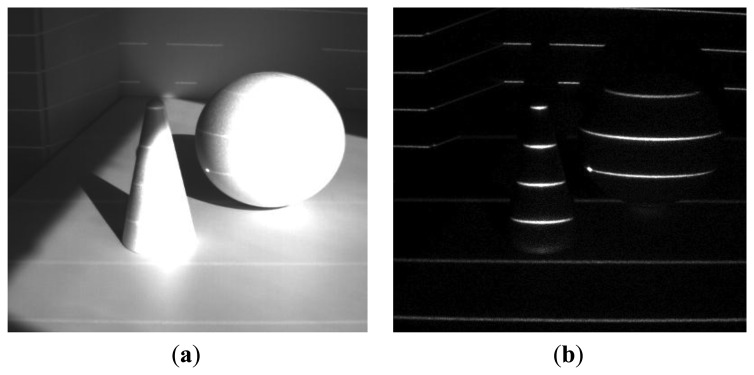
Background noise suppression: sunlight falling from the window. (**a**) Plane image; (**b**) demodulated image.

**Figure 8.**
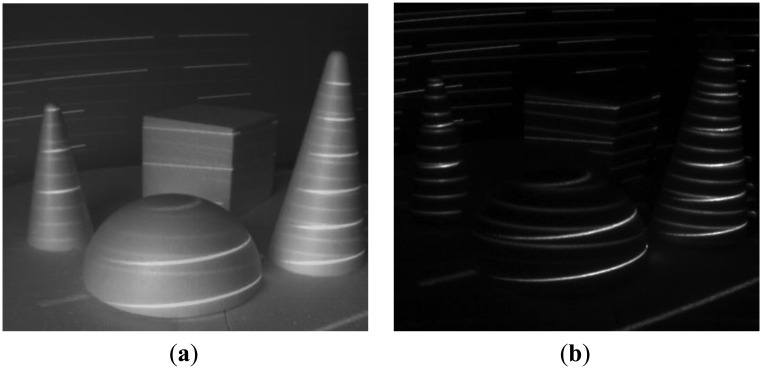
Interference suppression: interference of two identical devices having non-cyclic orthogonal modulation codes. (**a**) Plane image; (**b**) demodulated image.

**Figure 9.**
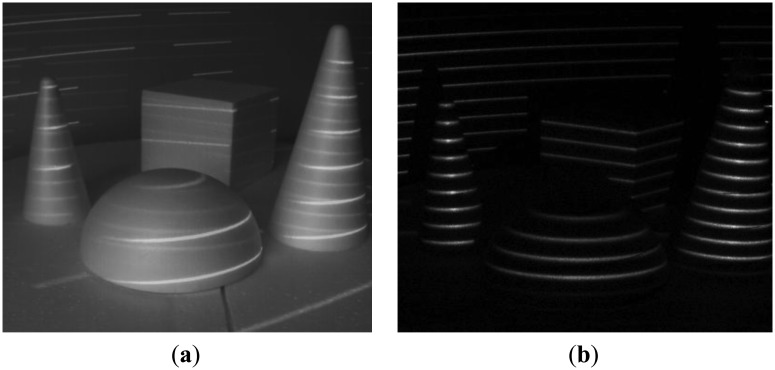
Interference suppression: interference of two identical devices with cyclic orthogonal modulation codes. (**a**) Plane image; (**b**) demodulated image.

**Figure 10.**
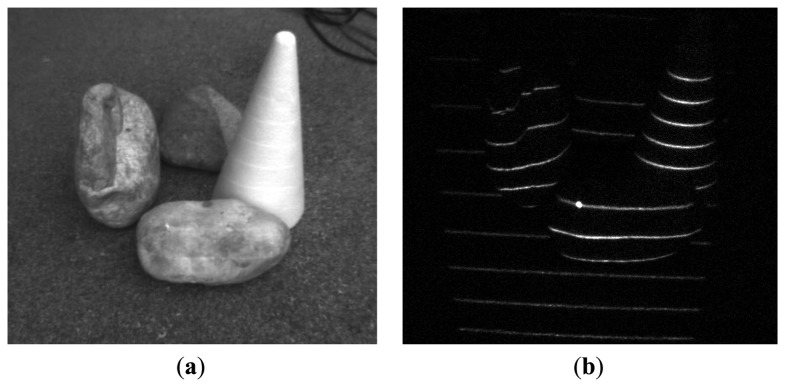

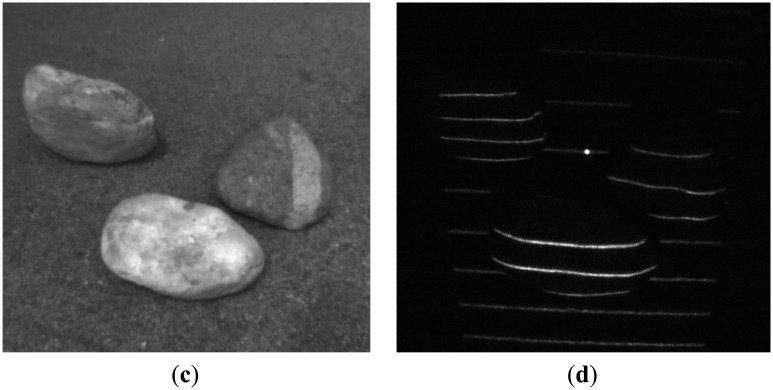
Background noise suppression: medium sunlight suppression outdoors. (**a**,**c**) Plane image, (**b**,**d**) demodulated image.

**Figure 11.**
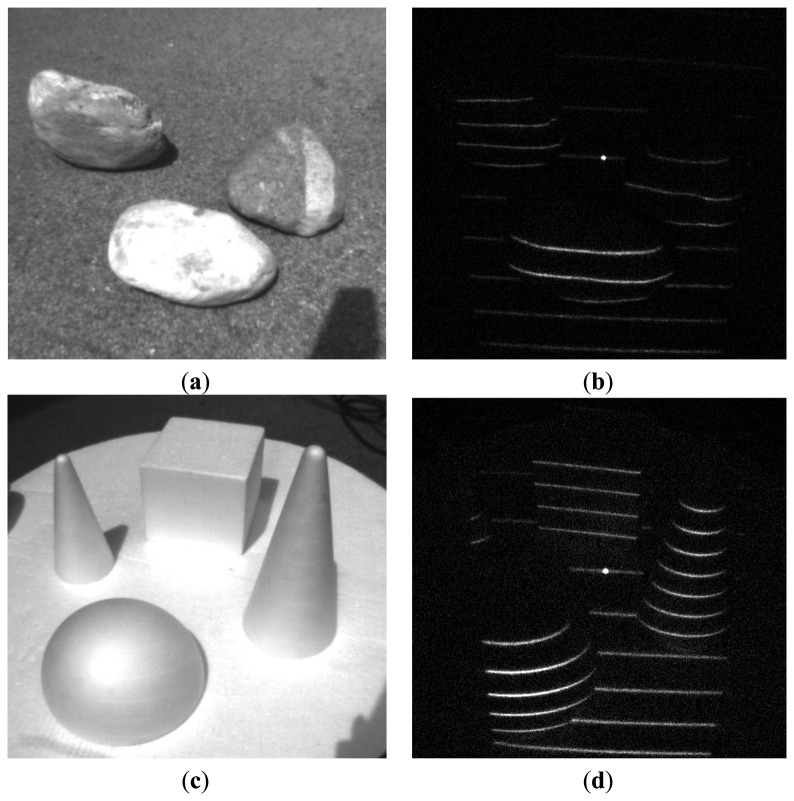
Background noised suppression: strong sunlight suppression outdoors. (**a**,**c**) Plane image, (**b**,**d**) demodulated image.

As it can be seen from the captured snapshots in Figure 5Figure 6, Figure 7, Figure 8, Figure 9, Figure 10 and Figure 11, our experimental ATRIS system demonstrates good results in closed environment and proves the feasibility of the suggested approach to compensation of the environmental factors outdoors. Even if laser projection is completely non-noticeable by the naked eye and much weaker than the interfering projection or environmental illumination, the modulation sequence can raise the level of signal pixels in the acquired distortion map. The proposed sets of cyclic orthogonal codes used as modulation sequences compensate the mutual interference as well as background noise and continuous-wave deliberations.

At the present state the described system setup has the following limitations caused by the sensor's characteristics, the setup design and its implementation:

1. The system's maximal frame rate is limited by the maximal sensor frame rate divided by *N*. Thus, when a longer modulation sequence is required, the frame rate degrades. This limitation can be eliminated by implementing a gated CMOS sensor capable of performing “in-pixel” demodulation.
2. The laser is triggered directly by the electronic shutter of the sensor and the duration of emission is limited by the sensor's exposure time. The low dynamic range of the sensor demands a reduction of the exposure time when capturing highly illuminated scenes. Since the laser power remains the same, the emitted laser energy is reduced proportionally to the reduced exposure time, so at durations of 0.01–0.02 ms the captured laser intensity level *l* becomes comparable to *δ*, which yields in increased background noise according to Equation (6). This limitation is especially noticeable with strong sunlight and reflecting surfaces, as shown in Figure 11(d).
3. The number of cyclic orthogonal codes of length *N* = 2*^k^* is limited by *k*. Overrunning this limit leads to the “frame collisions” effect demonstrated in Figure 8(b).

Since the discussed parameters are tightly correlated and directly affect the system's performance, future work will be aimed at finding an appropriate technique for adjusting them to dynamically changing scene conditions.

## 8. Conclusions and Future Work

Robust acquisition of spatial distortion maps represents the first step towards building a real-time ATRIS 3D vision system. An innovative approach to acquisition of projected patterns has been proposed and its performance evaluation has been presented in the paper. By introducing cyclic orthogonal codes as modulation sequences for sequential frame acquisition, the proposed computer vision system becomes robust to the environmental factors such as background noise, deliberate jamming and mutual interference of similar systems. The approach guarantees collision-free functionality for a limited number of users, which satisfies the requirements for multiple sensors operating in the same area. The system robustness to background light and co-located systems, its sensitivity and its dynamic properties can be adapted to application requirements by adjusting the length of the modulation sequence. The experimental setup of the system with 32-bit long sequence demonstrates the system's excellent performance in indoor environment and proves the suggested concept for outdoors scenes in direct sunlight.

In order to further improve the proposed approach and adapt it to a particular operating environment and system requirements, further work will mainly focus on finding the most appropriate pseudo random modulation sequences for a particular application. The length of the pseudorandom sequence is strongly correlated to the dynamic properties of the system, *i.e.*, the number of frames per second and the system robustness. The next challenge is in automatic adjustment of the system parameters to time-varying environment, *i.e.*, external illumination and number of interferers. This challenge includes methods for environment detection, quantitative evaluation of image quality and methods for adaption of the sequence length in order to find a tradeoff between the required frame rate and satisfying processing gain. In some applications a high density of proactive sensors in an area is expected, for example in automotive applications, where the number of users may exceed the number of available orthogonal pseudorandom sequences, which results in high mutual interference. Thus, an efficient technique should be found to decrease the mutual interference between systems. Among methods the pseudo random sequence hopping is a promising one, which has to be looked at. Finally, the introduction of the proposed approach to other proactive range detection methods can be studied.

## Figures and Tables

**Table 1.** Cyclic Orthogonal Codes selection table.

**Desired sequence length (*N*)**	4	8	16	32
**Number of codes in a set (*k)***	2	3	4	5
**Possible code sets (row indexes)**	2, r_2_∈ [3,4]	2, r_2_, r_3_∈ [5, …, 8]	2, r_2_, r_3_, r_4_∈ [9, …, 16]	2, r_2_, r_3_, r_4_, r_5_∈ [17, …, 32]
